# Recommendations for the measurement of sexual steroids in clinical practice. A position statement of SEQC^ML^/SEEN/SEEP

**DOI:** 10.1515/almed-2023-0020

**Published:** 2023-03-09

**Authors:** Gregori Casals, Roser Ferrer Costa, Eulàlia Urgell Rull, Héctor F. Escobar-Morreale, Jesús Argente, Gemma Sesmilo, Betina Biagetti

**Affiliations:** Servicio de Bioquímica y Genética Molecular, Hospital Clínic, IDIBAPS, CIBEREHD Universidad de Barcelona, Barcelona, Spain; Servicio de Bioquímica, Laboratoris Clínics, Hospital Universitari Vall d’Hebron, Universitat Autònoma de Barcelona, Barcelona, Spain; Servicio de Bioquímica, Hospital de la Santa Creu i Sant Pau, Barcelona, Spain; Servicio de Endocrinología y Nutrición, Hospital Ramón y Cajal, Universidad de Alcalá, Instituto Ramón y Cajal de Investigación Sanitaria IRYCIS y CIBER Diabetes y Enfermedades Metabólicas Asociadas CIBERDEM, Madrid, Spain; Departamento de Pediatría y Endocrinología Pediátrica, Hospital Infantil Universitario Niño Jesús, Universidad Autonoma de Madrid, CIBEROBN, Instituto de Salud Carlos III, Madrid, Spain; Servicio de Endocrinología y Nutrición, Hospital Universitari Dexeus, Barcelona, Spain; Servicio de Endocrinología y Nutrición, Hospital Universitari Vall d’Hebron, Universitat Autònoma de Barcelona, Barcelona, Spain

**Keywords:** estradiol, immunoassay, mass spectrometry, sexual steroids, testosterone

## Abstract

The proper clinical approach to a wide range of disorders relies on the availability of accurate, reproducible laboratory results for sexual steroids measured using methods with a high specificity and sensitivity. The chemiluminescent immunoassays currently available have analytical limitations with significant clinical implications. This position statement reviews the current limitations of laboratory techniques for the measurement of estradiol and testosterone and their impact on diverse clinical scenarios. A set of recommendations are provided to incorporate steroid hormone analysis by mass spectrometry in national health systems. International societies have recommended this methodology for a decade.

## Introduction

Steroid hormones are involved in individual development and maturation and have a role in a multiplicity of functions [1, 2]. Sex-steroid imbalance is associated with metabolic alterations, bone disorders, and the development/progression of some types of tumors, among other disorders.

Despite their crucial role, our capacity to measure steroid concentrations properly varies with the method.

In a broad range of clinical situations, it is necessary that steroid hormones are measured using techniques with high specificity, sensitivity, accuracy and precision, with accurate standardization. Ensuring that results are reliable is crucial for appropriate decision-making and prevents misdiagnosis, inappropriate treatment, and unnecessary monitoring.

This paper describes the techniques used for the measurement of steroid sex hormones and assesses their role in clinical practice. Consensus recommendations by the Spanish Societies of Laboratory Medicine (SEQC^ML^), Endocrinology and Nutrition (SEEN), and Paediatric Endocrinology (SEEP) are summarized.

## History of measurement techniques

The first steroid hormone techniques were based on chemical methods and bioassays. These methods had a limited analytical sensitivity (milligram or microgram per liter concentrations), and were mostly restricted to the analysis of conjugated steroids in urine. The advent of radioimmunoassays (RIAs) made it possible to measure steroid hormones with a higher sensitivity (nanogram or picogram per liter) and specificity in serum and plasma. The first RIA was developed in 1959 [3] and was initially designed for peptide hormones (insulin). Their use was later extended to smaller molecules such as steroids, being its use first extended to estradiol radioimmunoassay by Abraham et al. [4]. The emergence and implantation of RIA assays for the measurement of hormones has played a major role in the development of modern endocrinology.

The researchers that developed the first immunoassays for small molecules were aware of the difficulties and limitations of these techniques. The small size and low immunogenicity of steroid hormones made it necessary to develop additional optimized pre-analytical steps, which delayed the development of the first immunoassay for a steroid hormone. Steroid hormones are lipid molecules originated from cholesterol and released in limited amounts by enzymatic transformations, therefore, they are very similar in structure. These additional pre-analytical steps involved extraction with organic solvents and chromatography. These steps separated the different steroid hormones present in biological fluids such as serum, plasma and urine. This way, the first serum steroid assays were developed in the ‘70s, with good sensitivity and specificity. However, their use was constrained to specialized laboratories, given the time-consuming manual processing that samples required. The advantages of RIAs with preceding purification steps include the elimination of potentially interfering metabolites and denaturation of transport proteins such as sex hormone-binding globulin (SHBG) during extraction with organic solvents, which releases steroids (testosterone or estradiol). In RIAs, sample volume can be tailored to the optimal sensitivity. Moreover, different steroids can be measured in the same sample after separation by molecular weight and polarity using chromatography.

RIAs with preceding sample purification steps have contributed significantly to advances in endocrinology. In the field of clinical diagnosis, RIAs have been crucial for correct diagnosis and treatment and are still gold standard methods owing to their good analytical performance when adequately validated. However, these assays have some limitations, as they require radiation facilities, are time-consuming and are scarcely practical (it takes two days to measure a single steroid hormone in about 40 samples).

By the end of the ‘70s, the development of targeted antibodies and dual antibody techniques enabled the emergence of direct radioimmunoassays, which did not include a previous sample purification step. In the ‘80s and ‘90s, radioactive RIA labels were replaced with chemiluminescent, fluorescent or enzymatic labels. Coinciding with the substantial increase in demand for laboratory tests, these novel labels facilitated the incorporation of simpler and automatable reagent kits that enabled testing larger number of samples.

At present, direct chemiluminescent immunoassays (without previous sample extraction) are the most frequently used methods for the measurement of sex hormones. Although their analytical limitations were rapidly identified [5], it was not until 2000 that they became popular. This was due to the increasing evidence of the analytical performance of the existing methods, added to the increased availability of mass spectrometry-based methods. The study published by Taieb et al. [6] was a turning point in this field, as it revealed that of the 10 testosterone immunoassays evaluated, none had an appropriate analytical performance in the range of values frequently found in children and women. This finding challenged the clinical usefulness of the techniques in a variety of clinical scenarios [6, 7]. These findings in relation to testosterone determination were replicated in subsequent studies [8] and extended to other analytes such as estradiol. The studies by Lee et al. and Stanczyk [9, 10], which took mass spectrometry as a reference, demonstrated that immunoassays did not quantify serum estradiol concentrations in post-menopausal women in a precise and accurate manner.

In light of the difficulties to measure steroid sex hormones, including testosterone, with the required accuracy, reproducibility and sensitivity, in 2007 [11], the Endocrine Society recommended the use of mass spectrometry-based techniques as the method of reference for measuring circulating levels of testosterone [11]. Some years later, the Endocrine Society, along with the Centers for Disease Control and Prevention (CDC) released a consensus statement to standardize testosterone assays [12]. Next, CDC launched the Hormone Standardization (HoSt) Program [13], a program aimed at the standardization of testosterone measurements. The purpose was to improve the reproducibility, bias, total analytic error and comparability of testosterone assays. Finally, the year 2013 [14, 15] witnessed the development and validation of an LC-MS/MS assay calibrated to SRM 971, a traceable and commutable reference material prepared by the NIST (National Institute of Standards and Technology) for testosterone measurement. Surveillance within the framework of the CDC standardization program consistently demonstrates that mass spectrometry-based techniques are superior to immunoassays, although some improvements have been made to the latter.

On another note, there is cumulative evidence of the limitations of estradiol immunoassays. Hence, laboratories, scientific societies and manufacturers should work together to improve the existing analytical methods and facilitate standardization. The CDC HoSt program also includes serum estradiol measurement and regularly evaluates the performance of the different assays available [16]. There is robust evidence that mass spectrometry-based techniques have better performance, without significant improvements having been observed in the analytical performance of estradiol immunoassays [17]. The HoSt program was expanded to 25-hydroxyvitamin D, as the assays available for this analyte my present with some limitations similar than those observed in testosterone and estradiol.

## Pre-analytical considerations

Pre-analytical conditions are crucial for the measurement of analytes. Therefore, pre-analytical conditions must be highly controlled.

Secretion of testosterone follows a circadian rhythm, with values peaking at 8 a.m. and declining at 8 p.m. Variations of 36% have been reported [18]; therefore, in testosterone testing, blood should be drawn in the early morning. The majority of testosterone circulates bound to SHBG (high-affinity low-capacity binding) and albumin (low-affinity high-capacity), whereas a small fraction circulates unbound (free) reaching target tissues. Free testosterone converts to dihydrotestosterone within target cells, which binds to its cytosolic receptor, enters the cell nucleus, and binds to DNA-specific androgen response elements (ARE). For that purpose, the free fraction is more directly related to the action of dihydrotestosterone and is more relevant for clinical practice.

However, the accurate measurement of free testosterone is a challenge for clinical laboratories. Although there are commercially-available immunoassays, they are known to have significant limitations, and their use is not recommended in the clinic [19], [20], [21]. The gold-standard methods for measuring free testosterone include ultrafiltration and equilibrium dialysis. The use of these techniques enables free testosterone to be separated from bound testosterone before the RIA or mass spectrometry measurement. Nevertheless, these methods are technically complex and are not available in most clinical laboratories. Alternatively, it is recommended to determine free testosterone using equations that consider total testosterone, SHBG and albumin [22].

Regarding estradiol, ninety-five percent circulates bound to SHBG. Concentrations of estradiol in blood do not follow a circadian rhythm, although, as it occurs with testosterone in women, they change throughout the different phases of the menstrual cycle. For this reason, in women of childbearing age, blood must be drawn in the early follicular phase of the menstrual cycle.

## Challenges for analysis. Advantages and limitations of immunoassays

When optimal pre-analytical conditions are guaranteed, the clinical value of test results largely relies on the use of an appropriate measurement method. Measuring steroid hormones in serum for clinical and/or research purposes is challenging. Firstly, there are multiple structurally-similar endogenous and exogenous metabolites (cholesterol-derived molecules). Secondly, the range of concentrations of clinical interest is very wide, and finally, the presence of free and circulating protein-bound forms makes measurement difficult. Although immunoassays (IAs) are susceptible to some of these confounding factors, they are the most widely used methods. Of note, numerous scientific societies advocate that mass spectrometry-based methods should be used instead of IAs, as they are significantly more accurate and precise [11, 12].

An advantage of automated immunoassays is their simplicity and capacity to measure a high number of samples rapidly at a relatively low cost. Some of their limitations include the lack of specificity of the antibodies used, which may result in the overestimation of concentrations due to cross-reactivity with molecules similar to the analyte. Another limitation is the higher risk of differences due to the different matrixes used for calibrators and serum samples. Finally, automated IAs may show a higher difficulty in dissociating testosterone or estradiol from SHBG and, in general, have a limited sensitivity, which prevents the detection of steroid hormones at low concentrations.

An evaluation of the accuracy of immunoassays in testosterone testing using CDC LC-MS as a reference revealed significant inaccuracies for a concentration of 43.5 ng/dL (mean bias by manufacturers: Abbott Architect 30%, Beckman Coulter 83–89%, Siemens −8.5-22.7%, Roche Cobas 48%, Tosoh Bioscience 37%). The results reported for the different immunoassays for this same concentration, which is within the reference range for women, ranged from 34.4 to 87.8 ng/dL. Precision improved for higher values of testosterone (160 and 534 ng/dL), with <20% bias in most cases [23]. With respect to estradiol, popular commercially-available immunoassays (Siemens Centaur; Siemens IMMULITE; Abbott ARCHITECT; Roche Cobas) in male serum samples showed a positive bias of 11–74% for the 25th percentile (62 pg/mL), and 12–53% for the mean value (82 pg/mL) [24].

Dehydroepiandrosterone sulfate (DHEAS) has been identified as a potential interfering factor in testosterone immunoassays and suggested to cause overestimation [25]. Overestimation of testosterone is critical when relatively low concentrations are assayed. Such is the case of women, children, and men on follow-up for prostate cancer, in whom testosterone concentration is used as a surgical/chemical castration criterion [25, 26]. There are also cross-reactions with other endogenous metabolites such as 11-ketotestosterone or 11-hydroxytestosterone. Testosterone assay antibodies may also show cross-reactivity with synthetic steroids such as nandrolone, danazol, or norethisterone [27]. In the case of estradiol immunoassays, cross-reactions with aromatase inhibitors such as exemestane [28] due to their molecular similarity are of special relevance (androstenedione immunoassays may also yield falsely elevated concentrations by cross-reactivity with exemestane). This cross-reactivity has a considerable clinical impact since the effectiveness of aromatase inhibitors is assessed on the basis of estradiol levels. Cross-reactivity with estrogen receptor antagonists such as fulvestrant has also been described [29]. More recently, it has been described that oral estrogens may lead to falsely low concentrations of estradiol when measured by immunoassay [30]. The occurrence and degree of a specific interference in the analysis of steroid hormones depends on the antibodies used in that immunoassay, with significant differences among manufacturers.

## Contribution of mass spectrometry

Determination of these analytes should be based on methods free from interferences from other analytes (new therapies with monoclonal antibodies, biological drugs, etc.) with a similar structure or that interfere with the measurement procedure. Testing methods should also have sufficient sensitivity to be able to quantify low concentrations, as in specific physiological (in pediatric or female samples) or pathological settings.

**Figure 1:**
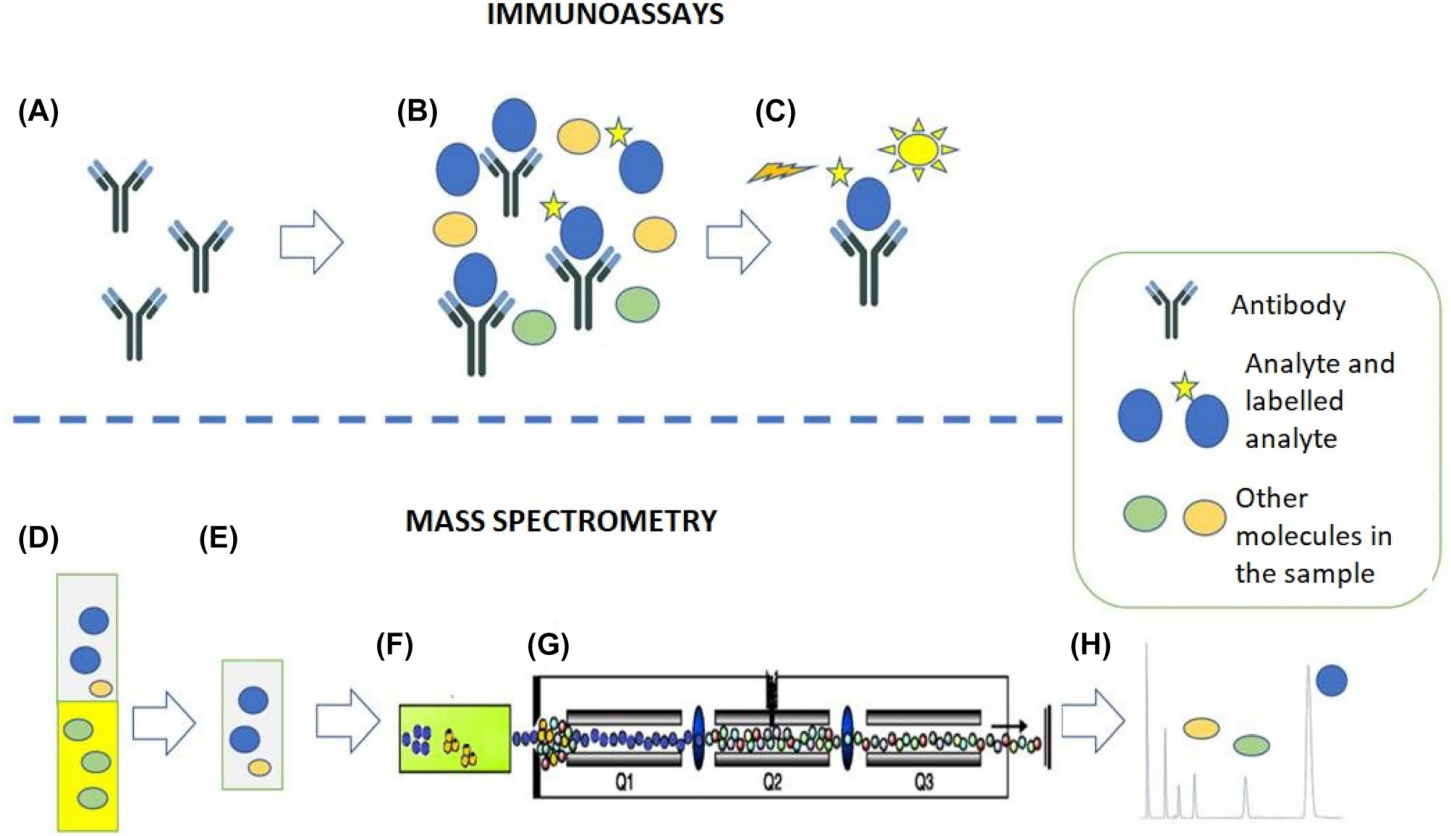
Immunoassays. (A–C) Example of competitive chemiluminiscence immunoassay. (A) By this method, a specific antibody recognizes the analyte of interest. (B) The antibody is incubated with the sample (containing the analyte of interest and other molecules) and with the labeled analyte. The analyte and labeled analyte compete for antibody binding sites. (C) The chemiluminiscence signal generated by the bound labeled analyte is recorded. In this example, the recorded signal will be inversely proportional to the amount of analyte initially present in the sample. (D-H) Mass spectrometry. Example of liquid chromatography tandem-mass spectrometry (LC-MS/MS). (D) and (E) Extraction of a serum sample using an organic solvent. This step eliminates potential interferences. (F) Separation of sample components by liquid chromatography. (G) Selection of analyte-specific ions. (H) Representation of results. The chromatographic peak area is directly proportional to the amount of the analyte initially present in the sample.

In this line, mass spectrometry makes it possible to overcome the limitations of immunoassays in a variety of clinical scenarios. The two techniques (immunoassay and mass spectrometry) have different fundamentals. Although there are multiple types of immunoassays and mass spectrometry assays, in general terms, the former are based on antigen-antibody reactions, whereas the latter directly rely on the structural characteristics (mass and mass spectrum) of the analytes (Figure 1).

High-impact journals increasingly require researchers to use hormone assay methods with appropriate performance, which also reflects the relevance of analytical performance in hormone testing. The Journal of Clinical Endocrinology and Metabolism announced in an editorial that the use of mass spectrometry will be required for the publication of research studies where steroid hormones are primary endpoints [31]. Some time later, the same Journal published a Letter of Concern about the complexity of hormone testing [32] and established the requirements for steroid hormone determination aimed at authors [33].

**Figure 2:**
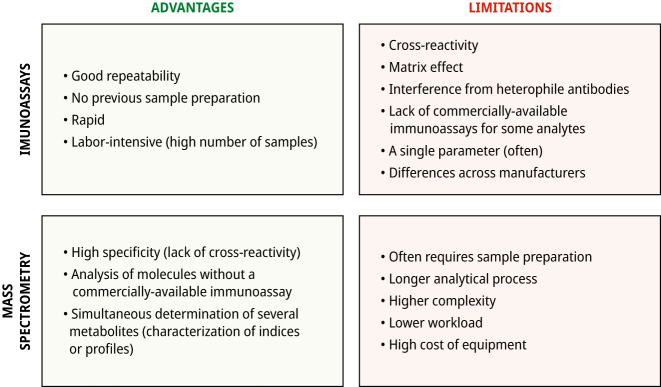
Advantages and limitations of immunoassay and mass spectrometry based methods.

**Figure 3:**
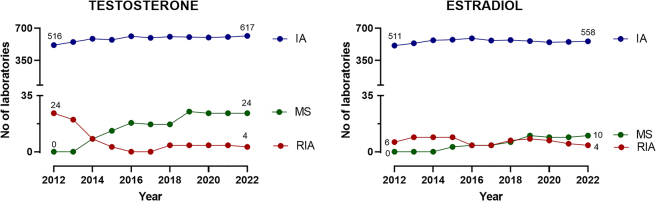
Number of laboratories that measure testosterone and estradiol by chemoluminiscent immunoassays (IA), mass spectrometry (MS), and radioimmunoassays (RIA) in accordance with the European External Quality Control Program of the Referenzinstitut für Bioanalytk, Germany.

Figure 2 compare the advantages and disadvantages of common chemiluminiscent immunoassays against mass spectrometry-based assays. Immunoassays are rapid and repeatable, which allows high workloads to be handled adequately. In contrast, mass spectrometry has a high analytical specificity (absence of cross reactions with endogenous or exogenous metabolites i.e. drugs [27], [28], [29]), has a lower matrix effect and is not affected by interference from heterophile antibodies. Other advantages of mass spectrometry include its versatility (it measures almost all analytes) or the capacity to measure several biomarkers simultaneously profiles or panels from the same sample. Its high specificity minimizes inter-laboratory variability, which facilitates the establishment of reference intervals or cut-off points for clinical decision-making. However, mass spectrometry requires the use of relatively costly equipment that requires considerable expense. This technique also requires previous sample preparation and is labor-intensive (highly-trained personnel is required), and has a longer turnaround time. These drawbacks limit the massive use of clinical laboratories. European external quality control programs reveal that the use of mass-spectrometry in clinical laboratories is increasing progressively (Figure 3). According to the UK National External Quality Assurance Service (UK NEQAS, year 2018), 15% of participating laboratories measure testosterone by mass spectrometry [34]. In Spain, experience with mass-spectrometry for the measurement of hormones other than steroids in the clinic is limited to the very recent last years.

## Clinical scenarios where mass-spectrometry is more useful

As mentioned above, steroid hormone assays must be sensitive, specific, accurate and precise for a wide range of concentrations [11, 12, 35, 36]. Mass-spectrometry is especially useful in a variety of clinical settings.

### Sensitivity: measurement of steroids at low concentrations

#### Measurement of estradiol at low concentrations

In special situations, such as surveillance of patients with breast cancer treated with aromatase inhibitors (when it is necessary to suppress endogenous estradiol concentrations). Assays must distinguish suppressed concentrations <1 pg/mL [37] from pre-treatment concentrations, which are 10–15 pg/mL for menopausal women. Likewise, women with other diseases such as endometriosis or leiomyomas receiving blocking and replacement therapies (GnRH agonist to induce medical castration, along with a low dose of estrogens) cannot be monitored appropriately due to the lack of a specific measurement technique with sufficient sensitivity for the expected low concentrations of circulating estrogens.

Elderly men and women have low concentrations of estradiol, ranging from 5–30 pg/mL. In some cases, the ability to measure low concentrations could be useful for patients such as men with prostate cancer receiving androgen deprivation therapy. In this setting, estrogen determination could help assess response to treatment in the bones and heart, and evaluate metabolic status [35].

To monitor puberal development in children, it is necessary to measure steroid hormones at low levels with high accuracy. This is of special relevance for prepuberal children and patients with early central, peripheral or mixed puberty. Defining normal concentration intervals during childhood is also important. In this stage of life, although rarely, some gonadal tumors release human chorionic gonadotropin thereby resulting in the production of sexual hormones often below the limit of detection of the method [35, 38], [39], [40]. Additionally, pubertal gynaecomastia in children, which causes an imbalance between estrogen and androgen activity cannot be distinguished with common immunoassays [41].

#### Testosterone testing

Most patients with polycystic ovary syndrome and other forms of androgen excess are not being adequately evaluated due to the lack of sensitivity and/or specificity of most immunoassays. Some studies have demonstrated that free testosterone correlates better with the clinical presentation of the polycystic ovary syndrome than total testosterone [42, 43]. However, as it was mentioned above, free testosterone, which gold standard method is based on ultrafiltration or equilibrium dialysis, is not available in common clinical laboratories. It is worth mentioning that formulas for the estimation of free testosterone require total testosterone to be measured by a precise and accurate method. In that case, performance of calculating formulas is similar to that of ultrafiltration or equilibrium dialysis based measurements. Since 2009, the Androgen Excess & PCOS Society recommends the use of liquid chromatography/tandem mass spectrometry as a gold standard for total testosterone testing. This Society also recommends calculating free testosterone using SHBG and albumin as the method of choice to estimate biochemical hyperandrogenism in these patients [44].

Determination of testosterone in women with reduced libido could be informative and is an unmet clinical need. Some studies, although controversial, suggest that female libido improves with testosterone replacement in women with hypopituitarism [45] or in ovariectomized premenopausal women [46]. However, current testing methods are not sufficiently reliable for testosterone concentrations after replacement therapy.

Other situations where high-sensitivity methods are required include the follow-up of patients with prostate cancer subject to chemical castration to suppress endogenous testosterone, during adolescence to evaluate early or delayed puberty, and at birth during the evaluation of mini-puberty in males.

### Specificity

Patients may have exogenous circulating estrogens/androgens such as steroid hormones from food, nutritional supplements, etc. [47]. Some of these compounds may have cross-reactivity with the immunoassay antibodies leading to misdiagnosis and the resulting expense and inconveniences for the patient derived from further unnecessary studies.

### Accuracy

Results should be comparable between laboratories. Reproducible data are essential for the analysis and control of patients whose assays are performed in different laboratories using different methods. The widespread use of properly standardized techniques would prevent patients on follow-up in a specific center to be referred to other centers due to a lack of inter-assay accuracy and reproducibility.

## Working group recommendations

In steroid hormone determination, mass spectrometry has demonstrated to be methodologically superior, especially when high sensitivity and specificity are required. Until all measurement techniques have not been standardized, it is recommended to use validated methods that meet CLSI reproducibility, sensitivity and accuracy criteria [48] for the population served. In addition, each laboratory should define its own reference intervals [49], [50], [51]. In this vein, the CDC HoSt program offers its services to laboratories worldwide at an affordable cost. We strongly recommend public clinical laboratories to comply with this program to evaluate the performance of their assays and to consider the replacement of those presenting poor performance with the standardized assays. This program also indicates the certified range of measurement for each method and provides valuable information for assessing usefulness in clinical practice. Although immunoassays may be somehow useful for measuring total testosterone in males with concentrations within the reference interval and estradiol in premenopausal women, studies from the last decade consistently recommend the use of mass spectrometry-based techniques for the appropriate determination of steroid hormones, especially.

(1) Testosterone: determination of serum testosterone in pediatric patients, women, and patients with hormone-dependent tumors should be performed using mass spectrometry-based methods with a high specificity and analytical sensitivity. Otherwise, it is recommended to consider using immunoassays with a good analytical performance as compared to a reference mass spectrometry-based method, such as the one assessed by the HoSt program [52].
(2) Estradiol: determination of serum estradiol in pediatric patients and women with breast cancer treated with aromatase inhibitors should be performed using methods with high sensitivity and specificity based on mass spectrometry in the initial evaluation and during treatment. The currently available immunoassays do not have adequate sensitivity in these scenarios, nor have they been granted HoSt certification (November 2022) [52].

We are aware that mass spectrometry-based methods are not available in all hospitals of the national health system. However, this Working Group recommends that these techniques should be available in at least one center of each regional health system, to be able to provide adequate support to the clinical situations previously described. This would also enable clinicians to acquire the experience required for achieving the progressive spread of the technique to other hospitals and adhere our national health system to international recommendations.
